# COLLABORATIVE CAPACITY BUILDING FOR STRENGTHENING REHABILITATION SERVICES IN SABAH, MALAYSIA: A PARTNERSHIP BETWEEN UNIVERSITY HOSPITAL AND STATE HEALTH DEPARTMENT

**DOI:** 10.2340/jrm.v57.40166

**Published:** 2025-02-05

**Authors:** Fatimah AHMEDY, Khin Nyein YIN, Syahiskandar SYBIL SHAH, Sukhbeer Kaur DARSIN SINGH, Nurul Afiqah Fattin AMAT, Frisca Aziah FRANCIS, Yusof IBRAHIM, Helen LASIMBANG, Rosalam SARBATLY, Kasim MANSUR, Candace GOH, Feroz KABIR

**Affiliations:** 1 Sabah Rehabilitation Research & Service Group, Faculty of Medicine & Health Sciences, Universiti Malaysia Sabah, Kota Kinabalu, Malaysia; 2 Department of Rehabilitation Medicine, Hospital Universiti Malaysia Sabah, Kota Kinabalu, Malaysia; 3 Department of Rehabilitation Medicine, Queen Elizabeth Hospital, Kota Kinabalu, Malaysia; 4 Department of Nursing, Faculty of Medicine & Health Sciences, Universiti Malaysia Sabah, Kota Kinabalu, Malaysia; 5 Dean’s Office, Faculty of Medicine & Health Sciences, Universiti Malaysia Sabah, Kota Kinabalu, Malaysia; 6 Director’s Office, Hospital Universiti Malaysia Sabah, Kota Kinabalu, Malaysia; 7 Board of Director's Office, Hospital Universiti Malaysia Sabah, Kota Kinabalu, Malaysia; 8 Physiotherapy Unit, Beaufort Hospital, Beaufort, Malaysia; 9 Department of Physiotherapy and Rehabilitation, Jashore University of Science and Technology, Jessore, Bangladesh

**Keywords:** capacity building, strengthening rehabilitation services, rehabilitation in community, academic partnerships

## Abstract

**Objective:**

This paper aims to outline the foundational framework for strengthening rehabilitation services in Sabah, Malaysia, through a collaborative capacity-building initiative between Hospital Universiti Malaysia Sabah (HUMS) and the Sabah State Health Department (JKNS). By focusing on academic, research, and service capacity-building, this partnership seeks to address the rehabilitation needs of the local population, particularly for individuals with functional limitations.

**Methods:**

The collaboration integrates HUMS’s academic and clinical strengths with JKNS’s existing rehabilitation services. Key components include developing postgraduate training for rehabilitation medicine, expanding community-based rehabilitation outreach services, and establishing a referral network between hospitals and community healthcare providers.

**Results:**

The partnership has resulted in the implementation of a comprehensive framework that enhances academic capacity, fosters research collaboration, and improves rehabilitation service delivery across Sabah. This approach is aligned with the WHO’s Rehabilitation 2030 initiative, advocating for stronger integration of rehabilitation into healthcare systems.

**Conclusion:**

The collaborative efforts between HUMS and JKNS demonstrate the critical role of partnerships between academic institutions and public health departments in strengthening rehabilitation services. This model offers a replicable strategy for influencing policy development and ensuring resource allocation to meet the growing rehabilitation needs in underserved regions.

## LAY ABSTRACT

This paper highlights the collaboration between Hospital Universiti Malaysia Sabah (HUMS) and the Sabah State Health Department (JKNS) to strengthen rehabilitation services in Sabah. The partnership focuses on training more rehabilitation professionals, conducting research to enhance services, and expanding care to rural areas. Through new training programmes, HUMS will increase the number of rehabilitation physicians in the state. The collaboration also implements a community-based rehabilitation model, ensuring people in remote areas can access care without the need for long-distance travel. This joint effort aims to make rehabilitation a core part of Sabah’s healthcare system, improving the quality of life for those recovering from serious health conditions or living with disabilities. By aligning with the World Health Organization’s global health goals, the partnership seeks to ensure better access to rehabilitation services, particularly in primary care settings, ultimately leading to better health outcomes for all.

Based on the World Health Organization (WHO) Global Burden of Disease study in 2019, approximately 2.41 billion people are estimated to have conditions that could benefit from rehabilitation services throughout the course of their illnesses (1). Functioning is a significant goal in rehabilitation, and impairment at any point on the continuum of care may contribute to the experience of disability affecting the quality of life (2).

In Malaysia, approximately 11.8% of adults live with some form of disability, a number that increases with age (3). Common health conditions leading to disability in the general population and elderly include non-communicable diseases (NCDs), stroke for instance, affecting 1.8 in every 1,000 people (3). Musculoskeletal conditions, such as osteoarthritis and lower back pain, affect over 20% of the elderly (3). Diabetes, affecting 18.3% of adults, is a major contributor to disability due to complications from neuropathy and foot ulcers (3). Additionally, neurodegenerative diseases such as dementia affect around 8.5% of those over 60 (3). Early detection and timely referral to rehabilitation services could mitigate long-term disability, but this requires well-coordinated efforts within the healthcare system.

In Sabah, a state in East Malaysia, the challenges of disability are compounded by both geographic and infrastructural barriers. According to the Department of Statistics Malaysia (DOSM) Household Income and Expenditure Survey in 2022, Sabah has a poverty rate of 19.7%, and approximately 1.6 million people (over 40% of the state’s population) reside in rural areas, making access to healthcare even more challenging (4). The diversity in geographical landscapes and the limited number of healthcare facilities restrict early intervention and access to rehabilitation services. A study conducted in rural communities in the northern region of Sabah found that while road access has improved, many inhabitants do not own private vehicles, and public transportation is scarce, often requiring walking distances of several hours (5). Only 56% of the population has access to primary healthcare facilities within a 5-km radius, and rehabilitation services are significantly lacking, with just 1.5 rehabilitation physicians per 500,000 people (5).

This shortage of trained professionals and resources exacerbates the situation, leaving individuals with disabling conditions and injuries without timely care, resulting in prolonged disability. Understanding the needs of the people of Sabah is critical in ensuring rehabilitation awareness and services are accessible throughout the state. It is essential to foster a collaborative network, appreciate key rehabilitation care providers’ roles, and implement sustainable strategies that address these gaps to ensure equitable access to rehabilitation services in the state.

## Establishment of Universiti Malaysia Sabah (UMS) as the only public university in Sabah

Three years after its establishment, UMS marked a significant milestone in the development of medical education in Sabah, as it became the only public university under the Ministry of Higher Education (MOHE) of Malaysia to train medical undergraduates in that state since 1998. Through the Faculty of Medicine and Health Sciences (FMHS), UMS initiated its Doctor of Medicine (MD) programme to address the increasing demand for medical professionals in the region, particularly in underserved and rural areas. The programme integrates both academic learning and clinical practice, ensuring that graduates are well prepared to meet the healthcare needs of the local population. The university’s contribution to medical education has profoundly impacted the Sabah state healthcare system, producing a steady stream of qualified doctors who serve in various hospitals, clinics, and health centres throughout the state.

In 2018, UMS expanded its healthcare education portfolio by launching a postgraduate Doctor of Public Health programme that trains specialists to address broader population health challenges. This development was a response to the rising need for public health experts to manage emerging healthcare issues such as infectious diseases, non-communicable diseases, and health disparities in rural communities. The programme equips graduates with the skills necessary for disease prevention, health promotion, and health policy development, making them critical contributors to the advancement of public health infrastructure in Sabah. With its dual focus on training both medical practitioners and specialists, FMHS continues to play a pivotal role in shaping the future of healthcare in the state through education programme development and certifications.

## Development of Hospital UMS (HUMS) as the sole university hospital in Sabah

HUMS is the eighth university hospital established under the Ministry of Higher Education (MOHE), strategically located in Kota Kinabalu, the capital of Sabah. The construction of this facility was initiated in 2019 to meet the growing need for a comprehensive teaching hospital dedicated to serving the region’s academic, research, and healthcare service needs. The hospital was developed in response to the increasing demand from both undergraduate and postgraduate students at the FMHS, who required a robust platform for clinical training and professional development. Its establishment aims to strengthen the academic and practical components of the medical curriculum, ensuring that students receive a well-rounded education that prepares them for the complexities of modern healthcare. Moreover, HUMS is envisioned to enhance the healthcare infrastructure in Sabah, offering specialized services to the local population while fostering a culture of research and continuous improvement in medical sciences.

In line with the hospital’s vision as the leader in advancing healthcare and wellness through empowerment, particularly in the Borneo region, HUMS is dedicated to training top-tier healthcare providers and improving access to innovative, compassionate, comprehensive, and collaborative global standard healthcare. The hospital, with over 50 departments, aims to be the primary training facility for teaching and learning of postgraduate medical curriculum. The Department of Rehabilitation Medicine of HUMS is one of the 15 clinical-based departments in this hospital and started operating comprehensive outpatient services since 2020. The department aims is to deliver evidence-based rehabilitation medicine practice and high-quality technology-driven services. Through multidisciplinary teamwork consisting of rehabilitation physicians, physiotherapists, occupational therapists, rehabilitation nurses, and speech and language therapists, it aims to benefit people with disability, individuals with disabling conditions, and those experiencing some degree of disabilities, holistically and by promoting social inclusivity.

HUMS is entrusted with a high-budgeted community-based programme as a direct initiative from MOHE (*Kementerian Pendidikan Tinggi* [KPT] in Malay), namely the KRIS programme. KRIS stands for *KPT Prihatin, Komuniti Sejahtera*, translated as Caring MOHE for a Prosperous Community. This programme serves as a platform to bring health promotions and services from university hospitals across the nation to rural residents of the country (6). The coverage of the communities is based on the location of the hospital university, in which HUMS is tasked to conduct the programme within the state of Sabah and at the time of writing there have been almost 130 engagements since its launch back in mid-2021. Unpublished data from HUMS has demonstrated that KRIS programme has reached more than 8,000 individuals with the involvement of more than 10 specialties throughout 25 rural districts of Sabah.

In addition, HUMS has a strong partnership with the Social Security Organization (SOCSO), a government-linked agency (GLC) as one of the key stakeholders in provision of rehabilitation services for employed individuals (*Orang Berinsuran* [OB] in Malay) who have made insurance contributions to the organization during their employment (7). The partnership was initiated in 2022 with a Memorandum of Understanding (MoU) and the collaboration is not only confined to delivering rehabilitation care to the OBs but also in establishing a network on disability management that is highly relevant to rehabilitation medicine.

## Sabah State Health Department as the key healthcare provider in Sabah

Sabah State Health Department (*Jabatan Kesihatan Negeri Sabah* [JKNS]) is responsible for healthcare services throughout the state and falls under the purview of the Ministry of Health (MOH), Malaysia. Its main objective is to implement the ministry’s healthcare programmes at the state and district levels to improve and maintain a standard of health, enabling its people to lead productive economic and social lives. Its biggest mission is to provide preventive measures, effective treatments, and rehabilitation for the less fortunate groups, especially for a state with more than 70% of its land within rural areas.

Since 2008, JKNS has introduced rehabilitation services for Sabah at Queen Elizabeth Hospital (QEH) as the main tertiary hospital located in the capital city of the state. Fifteen years later, Rehabilitation Services of Sabah offers additional comprehensive rehabilitation care at 2 more hospitals, namely the Duchess of Kent Hospital (HDOK) in Sandakan, and Keningau Hospital. These 3 hospitals have in-house rehabilitation physicians catering for both inpatient care and outpatient services while providing outreaching services to 19 district hospitals in the rural areas of the state, specifically as outpatient rehabilitation specialist clinics. In each of these district hospitals, the JKNS has allocated human resources for running physical and occupational therapy services. However, rehabilitation services in Sabah are confined to hospital-based care, placing strain on the primary care services to increase awareness and competency in providing rehabilitation care within community settings.

Under the Public Health Services of JKNS, a specialized service called the Domiciliary Care Service was introduced by MOH in 2001 (8). The service aligns with the health transformation needed and is characterized by good health services that are close to the community. The Domiciliary Care Service provides holistic care and is intended for patients with disabling conditions who are bedbound, requiring continuity of nursing and rehabilitation supervision after discharge from government hospital facilities. This service is provided by elected health clinics through a multidisciplinary team home service at interval visits for a total of 3 months’ duration upon discharge. In Sabah, less than 10 health clinics provide such a service, limiting the coverage area for patients to receive such care, The home visit team members involved in this care are physiotherapists, occupational therapists, nurses, and occasionally dietitians, reflecting the scarcity of rehabilitation physicians in Malaysia who offer the prospect of reaching out to the community beyond hospital care. Furthermore, those with a disability or disabling conditions who are more independent, with the potential for functional recovery, are not eligible for the Domiciliary Care Service and must consider logistical support to receive rehabilitation services at district hospitals. Hence, there is a need to strengthen capacity building in delivering comprehensive rehabilitation service provision within community settings to broaden the scope of outreach.

## Addressing the needs for strengthening rehabilitation services in Sabah

In response to the recent World Health Organization (WHO) guidelines on rehabilitation, the World Health Assembly Resolution on Strengthening Rehabilitation in Health Systems provides a critical framework for improving rehabilitation services globally (9). This resolution highlights the need to integrate rehabilitation into national health systems as a core service, addressing the increasing global demand for rehabilitation due to ageing populations and the rising prevalence of non-communicable diseases. By leveraging this resolution, local and national stakeholders in Sabah can strengthen advocacy efforts to prioritize rehabilitation in healthcare policies and ensure adequate resource allocation. The resolution serves as a powerful tool for aligning national rehabilitation efforts with global health priorities, facilitating a more structured and evidence-based approach to building capacity, expanding workforce development, and improving service delivery. Incorporating the principles of this resolution into Sabah’s health strategies could significantly bolster the region’s advocacy for rehabilitation, influencing policy reforms that emphasize access, equity, and quality of rehabilitation services.

Malaysia has less than 5 rehabilitation physicians per 1 million people, approximately and, at the time of writing, Sabah has 3 rehabilitation physicians per 1 million people (10). These figures, although recorded as slightly higher than the average ratio of rehabilitation physicians in Southeast Asian countries (less than 2 rehabilitation physicians per 1 million people), remain far from sufficient for Sabah state and the Malaysian context, with less than 160 active rehabilitation physicians in the whole country, across government and private sectors (11). It is recommended for allied healthcare professionals, i.e., physiotherapists and occupational therapists, to be within the recommended figure of 1 therapist per 1,500 people. Malaysia, with more than 30 million people, has approximately 5,000 physiotherapists and 2,500 occupational therapists registered in corresponding professional practice bodies, a staggering low figure to cater for the entire country.

The World Health Assembly passed a significant resolution in mid-2023, co-sponsored by 20 countries, focusing on strengthening rehabilitation within health systems (9). The resolution emphasizes expanding and integrating rehabilitation services as part of Universal Health Coverage (UHC), underscoring the essential role of rehabilitation in primary care and its importance in emergency preparedness and response efforts. From the perspective of academic capacity building of relevant professions in the rehabilitation field, there are only 2 national training facilities that are accredited to produce competent rehabilitation physicians and recognized by the National Specialist Register (NSR) of Malaysia, namely the University of Malaya and Universiti Teknologi MARA, which are in the Klang Valley of Malaysia in the Peninsular region. Those who have received training through parallel pathways such as Rehabilitation Medicine Fellowship training under the Australasian Faculty of Rehabilitation Medicine (AFRM) are also recognized by the NSR, but such training requires trainees to be completely placed in other countries, unlike those who have the privilege of being certified while practising in Malaysia, for instance through the MRCP examination. In a broader aspect for allied healthcare professionals, MOH has implemented structured training at diploma level for physiotherapy and occupational therapy programmes across the country, for which the JKNS is tasked to conduct the programmes in Sabah. However, there are less than 10 higher education institutes accredited to deliver bachelor’s degree programmes for these professions. Only 3 programmes are available at public universities and the remainder are available at private institutions imposing significant educational fees.

Viewed through the lens of public health, the increasing prevalence of various disabling health conditions in the population, including those affected by ageing, warrant proper screening methods by primary care practitioners for referral thresholds to a rehabilitation team. Primary care practitioners in Malaysia usually comprise family physicians (equivalent to general practitioners), medical officers, and nurses in local health clinics, and they play a pivotal role in detecting early signs of disability, especially among the elderly. In Malaysia, the shortage of healthcare professionals is a growing concern, with a 1.6 per 1,000 population ratio for family physicians, and even fewer in rural areas like Sabah, where this can drop to as low as 1 physician for every 2,000 individuals (10). This shortage, combined with long waiting times and logistical issues, exacerbates the delayed access to crucial rehabilitation services, particularly for ageing and disabled populations.

Henceforth, a strategic collaborative effort must be forged between key players in rehabilitation care in the state to fulfil these identified gaps within the next 5 years, not only as a means to respond to the WHO 2030 call, but to forge the path that will form a sustainable rehabilitation network for impactful outcomes. The main objective of this paper is to highlight the foundation framework in Sabah state for addressing the need to develop comprehensive and system-wide rehabilitation services across the entire continuum of care. We aim to demonstrate the strategies and ongoing efforts of capacity building on education, research, and services for strengthening rehabilitation services in Sabah through collaborative efforts between HUMS and the Sabah State Health Department.

## METHODS

Since the establishment of FMHS and HUM, both have actively collaborated with JKNS to advance healthcare education, research and services in the region. A pivotal step in this partnership was the signing of an MoU in 1996, coinciding with the initiation of the Doctor of Medicine (MD) programme at what was then known as the School of Medicine & Health Sciences. This MoU was designed to foster capacity building in both teaching and learning for undergraduate medical students and nursing diploma students, integrating practical training with JKNS’s extensive healthcare network across Sabah.

Over the years, this collaboration has strengthened the academic and professional development of medical and healthcare students, with ongoing contributions to enhancing the quality of healthcare delivery in the state. From 2019 onwards, formalized discussion ensued between FMHS, HUMS, and JKNS with regular sessions for brainstorming on training coordination, learning progress, and quality improvement for implementation of teaching, learning, and assessments of students. This partnership continues to serve as a cornerstone for joint efforts in medical education, ensuring that future healthcare professionals are well prepared to meet the region’s evolving healthcare needs, including emerging needs for rehabilitation practitioners.

In the years since 2020, JKNS has served as a key partner for a joint committee with HUMS to establish a solid partnership towards developing a smart hospital university that is tasked to deliver advanced care to the people of Sabah. The Department of Rehabilitation Medicine of QEH is one of the integral members on this committee with a designated advisory role for HUMS in facilitating the setting up of a rehabilitation medicine department.

Through this collaborative network between FMHS, HUMS, and JKNS, a structured foundation framework is being constructed, aiming to establish a robust, sustainable, and well-integrated rehabilitation service network in the state. The following key frameworks are included in 5-year planning from 2020–2025.

### Developing a postgraduate rehabilitation medicine curriculum

The mission is to establish a dedicated postgraduate medical curriculum focusing on rehabilitation medicine within UMS as the sole provider of training in the Borneo region. This programme aims to produce qualified rehabilitation physicians who can lead and enhance rehabilitation services in Malaysia, particularly for Sabah. The curriculum should cover comprehensive training in physical and rehabilitation medicine that covers hospital care, transitional follow-up, and community outreach, with clinical rotations set in both HUMS and JKNS facilities. Close collaboration with JKNS will ensure the curriculum addresses the specific rehabilitation needs of Sabah’s population.

### Developing an undergraduate allied healthcare professionals curriculum

The emphasis is to develop academic programmes at degree level for allied healthcare professionals, focusing on physiotherapists and occupational therapists. These programmes are designed to fulfil the needs of the MOH, in which hundreds of diploma graduates are required to enhance their competency level due to the urgent needs based on the national call to upgrade these professionals to at least degree level in recognition of their expertise. UMS will fulfil these needs, and the programmes developed will adhere to international standards that ensure graduates are highly skilled in delivering rehabilitation care. Emphasis will be placed on both theoretical knowledge and hands-on clinical training, with practical placements across rehabilitation units in HUMS and JKNS facilities.

### Joint training programmes for rehabilitation practitioners

The focus is to implement ongoing professional development programmes for rehabilitation practitioners across Sabah state through partnership. This includes expansion of clinical training opportunities for rehabilitation professionals, medical, nursing, and allied health students by utilizing the rehabilitation facilities at JKNS facilities and HUMS. These structured clinical placements will provide real-world experience, mentorship, and the opportunity to apply their academic knowledge in practical settings, preparing them for professional roles in rehabilitation care and enhancement of their competency skills. Similarly, professional development programmes will include workshops, seminars, and certification courses, focusing on updated rehabilitation techniques, evidence-based practices, and interdisciplinary collaboration. The goal is to equip rehabilitation professionals with the right competencies and skills to deliver high-quality rehabilitation care in a variety of settings throughout the state, from well-equipped rehabilitation facilities in the urban vicinity to limited resource centres in remote areas.

### Establishing a rehabilitation referral network

The objective is to create a referral network within JKNS facilities, and between JKNS and HUMS for improvement in the coordination of rehabilitation care. The government hospitals under JKNS deliver excellent core rehabilitation services and the MOH has implemented clustering of hospital systems nationwide since 2022. This system places several smaller district hospitals within the vicinity in a cluster, with 1 larger district hospital as the tertiary centre housing various experts from specialties including rehabilitation medicine. For Sabah state, there are a total of 6 clusters excluding QEH and HDOK, and each is planned to have at least 2 in-house rehabilitation physicians for running both inpatient and outpatient rehabilitation services. Additionally, HUMS complements the existing and planned rehabilitation services by providing cutting-edge advanced rehabilitation care in the state. The hospital university is equipped with transcranial magnetic stimulation, bodyweight-supported gait training, AI-generated pain relief modalities, and will be receiving more advanced technologies once the hospital is fully operational by January 2026. Beyond hospital settings, the local health clinics are responsible for the continuum of care of the patients. This network will facilitate efficient communication, patient transfers between different levels of care, and transitioning from hospital care to the community, ensuring timely access to rehabilitation services.

### Rehabilitation in community outreach

The purpose is to strengthen community outreach efforts by developing and implementing rehabilitation programmes, conducted by the community through the joint efforts of HUMS and JKNS. These programmes will focus on engaging local communities in the rehabilitation process, raising awareness of the importance of rehabilitation, and training community healthcare workers and volunteers in providing basic rehabilitation treatments. Thes approach to fostering active collaboration between healthcare professionals and community members will improve access to rehabilitation services, particularly in underserved rural areas. In addition, primary care practitioners and healthcare personnels will be trained to triage people in the community attending local health clinics for early identification of the need for referral to rehabilitation.

### Initiating collaborative research

The goal is to foster joint research initiatives between HUMS and JKNS in line with the Rehabilitation 2030 initiative, which highlights the importance of strengthening health systems to provide rehabilitation as the critical aspect to fulfil the unmet need for rehabilitation worldwide. Research topics should incorporate rehabilitation care training, technology-enhanced rehabilitation care, and novel therapeutic interventions aimed at improving patient outcomes with the main emphasis on the health system as the pillar to support the initiatives. Although the opportunities for research in rehabilitation are vast due to the nature of disease heterogeneity and the availability of proven techniques, the collaboration will concentrate on investigating effective methods that can improve patient outcomes at population level in the context of Sabah. The usability of moderate technology with remote access for patient monitoring will be implemented through living laboratory approach.

## RESULTS

Based on this laid foundation framework, several programmes and activities are being initiated with ongoing execution. The key players who lead the collaborations between these partners are the heads of departments from both QEH and HUMS, with excellent support from the top management of FMHS, HUMS, and JKNS.

### Postgraduate rehabilitation medicine curriculum development

Seeing the need to strengthen capacity building in providing rehabilitation services in Sabah, the university has established a more concrete collaboration with JKNS. In 2021, FMHS appointed the heads of services across various medical and health specialties in JKNS as strategic partners of corresponding departments of HUMS. This includes the appointment of the Head of Rehabilitation Service of Sabah as a key partner to plan advancement of the academic, research, and rehabilitation medicine services of the state in collaboration with UMS. In the same year, the faculty had formed a board of studies consisting of relevant stakeholders and experts from the Department of Rehabilitation Medicine of QEH, other hospital universities in Malaysia, and members of the Conjoint Board of Rehabilitation Malaysia to construct a postgraduate rehabilitation medicine training curriculum in UMS. The curriculum is planned as a 4-year training programme equivalent to doctorate level with an entry requirement confined to those with MD (or equivalent) degree. It has been approved at the faculty level and received positive response from the conjoint board. Following this, the curriculum must be vetted by several academic authorities before it can be endorsed at MOHE and receive candidates for rehabilitation medicine specialty training. The training facilities at QEH for this programme have already approved and accredited by the conjoint board.

### Undergraduate allied healthcare professionals curriculum development

FMHS initiated engagements with leaders of physiotherapy and occupational therapy bachelor programmes from Universiti Teknologi MARA in 2020 for further advice on developing and implementing similar academic curricula at FMHS. However, the major drawback to progress the low number of academic staff in the state from physiotherapy and occupational therapy backgrounds with recognized master’s and doctorate qualifications. Therefore, the right strategic plan to overcome this is to increase capacity building among local physiotherapists and occupational therapists through PhD enrolment. Once the minimal number of academic staff for the programmes is achieved, the next step is to develop the curriculum for these 2 programmes. This can only be expected to progress from 2027 onwards and will require the combined training facilities of QEH and HUMS.

### Joint training programmes for rehabilitation practitioners

QEH and UMS have collaborated several times in organizing conferences and workshops on rehabilitation, at both state and national levels. The first local-level collaboration was initiated in 2019, focusing on stroke rehabilitation, followed by spinal cord injury rehabilitation at international level in 2021 and the 15th Annual Malaysian Rehabilitation Medicine Conference in 2024. In addition, since 2022 HUMS has collaborated with JKNS for clinical attachments of its staff at QEH and other tertiary hospitals of the state. This will provide the necessary training opportunities for HUMS staff in preparation for operational aspects of delivering medical and healthcare services in a hospital setting. The Department of Rehabilitation Medicine of HUMS has deployed more than 5 of its staff so far to QEH, in turn, from each of the physiotherapy, occupational therapy and nursing units, which has improved their competencies in respective fields.

### Rehabilitation referral network establishment

**Fig. 1:**
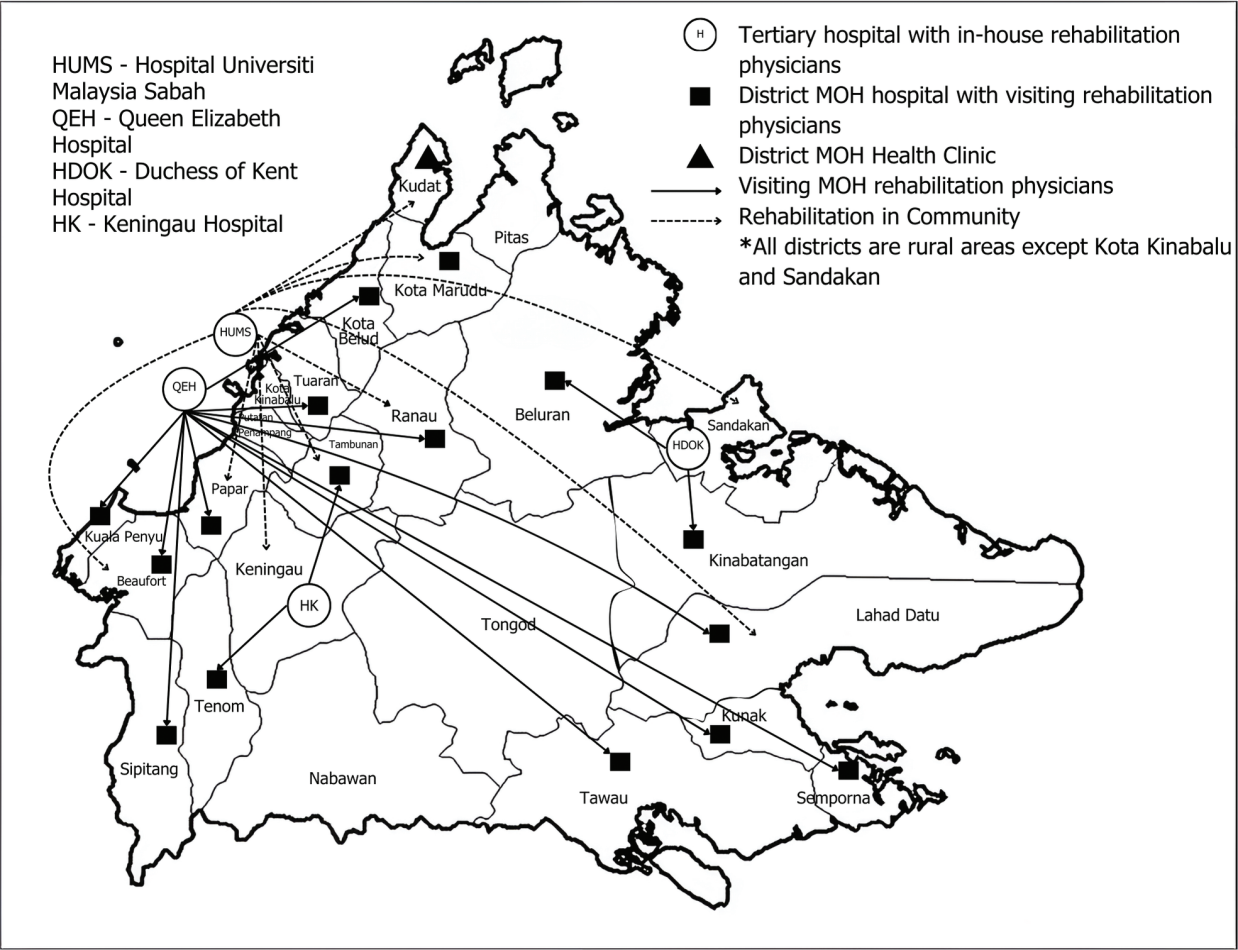
Network of rehabilitation services in Sabah state by HUMS and JKNS.

The collaboration between the Sabah State Health Department (JKNS) and its hospitals, with support from Universiti Malaysia Sabah (UMS), in establishing the Rehabilitation Referral Network aims to improve access to rehabilitation services across the state (Fig. 1). Queen Elizabeth Hospital serves as the main facility providing inpatient care for patients from the west, north coast, and midland regions of Sabah, while the Duchess of Kent Hospital in Sandakan caters to those from the east coast. In addition, UMS not only addresses the rehabilitation needs of its staff and students as well as public clients, but also plays a critical role in the network by receiving referrals from JKNS hospitals. This is due to the university hospital’s access to advanced technologies such as repetitive transcranial magnetic stimulation (rTMS), transcranial direct current stimulation (tDCS), bodyweight-supported gait training equipment, and the Pain Bot machine (a pain detection and treatment device), which are utilized for neuromodulation therapy in conditions like stroke and traumatic brain injury, and many musculoskeletal disorders.

Once patients are discharged from the rehabilitation ward, patients are referred to outpatient services that are available in various district hospitals throughout Sabah, ensuring continuity of care, including the ongoing Domiciliary Care Service under the JKNS. However, the majority of these services are confined to physiotherapy and occupational therapy aspects that operate with basic rehabilitation equipment and tight outpatient scheduling. Therefore, HUMS engaged with a local stroke community in Beaufort of Sabah and community leaders in that area in 2023 to understand their needs regarding rehabilitation care in the community. Three key issues were highlighted: (*i*) the logistical barrier on accessible transport for attending outpatient therapy sessions due to the large coverage area for the hospital to cater to and the geographical barrier, (*ii*) tight scheduling secondary to a limited numbers of therapists, and (*iii*) a lack of rehabilitation education and awareness sessions within the community. The community leaders highlighted the importance of empowerment of their people so that basic rehabilitation care can be provided in a sustainable manner in addition to the regular follow-ups with the rehabilitation team to monitor progress. Herein lies the importance of adding rehabilitation outreach in communities in the referral network.

### Rehabilitation in community outreach

The Department of Rehabilitation Medicine in UMS runs a specialized outpatient rehabilitation service, the Rehabilitation in Community model, which aligns with the MOHE’s wish to transform healthcare for the nation. This model represents an innovative framework for improving access to and the quality of rehabilitation services by bringing multidisciplinary expertise directly to communities. It is being developed to be part of the broader healthcare continuum, aimed to ensure that rehabilitation care is accessible even in remote or underserved areas, which is particularly relevant in geographically challenging regions of Sabah. Key elements of this model include a multidisciplinary approach, structured care and monitoring, awareness and education, and fostering local synergy to enhance the effectiveness and sustainability of rehabilitation efforts. This service model is currently conducted on a regular basis at Sikuati Health Clinic of MOH, situated in the northern part of Sabah within the district of Kudat. Primary care practitioners including a family physician (which is equivalent to general practitioner), medical officer, and nurses refer their patients to rehabilitation team members of HUMS based on the diagnosis and the need for more specific interventions. The cornerstone of this model is the involvement of a multidisciplinary team composed of rehabilitation experts who actively reach out to communities. This team encompasses a rehabilitation physician, physiotherapist, occupational therapist, and rehabilitation nurse. One of the unique features of the model is its focus on delivering structured rehabilitation care with ongoing monitoring. This ensures that patients are not only treated initially but are also followed up through continuous assessments and evaluations to track progress and adjust treatment plans accordingly. The model adopts a dedicated outpatient scheduling system, wherein specialists conduct outreach visits once a month and therapists provide follow-up care every 2 weeks. In addition to direct patient care, the model emphasizes the importance of awareness and education within communities. Rehabilitation teams promote preventive care and provide health education on issues such as obesity management, diabetic foot care, fall prevention in the elderly, and managing neurodegenerative conditions like dementia and Alzheimer’s disease. These awareness campaigns not only empower patients and caregivers but also foster a preventive approach to health, reducing the burden of chronic diseases that often require rehabilitation services. A significant strength of this model lies in its ability to create local synergy between healthcare providers and local community actors. By working closely with the local health clinic, the rehabilitation teams ensure that referrals are streamlined, and care is coordinated across different levels of the healthcare system.

The department also actively participates in the KRIS programme with its own rehabilitation health screening and consultation. However, the model is not implemented in the KRIS programme due to the nature of its sessions, which emphasise the awareness aspect, rather than delivery of care. However, our experiences through engagement with the KRIS programme have noted a significant gap, i.e., the lack of rehabilitation services in primary care for those residing remotely. More than 65% of the community in rural areas who have attended our screening and consultation booth were either yet to be assessed by any rehabilitation team for underlying disabling conditions or have a lack of awareness of rehabilitation services. Therefore, HUMS is partnering with QEH in the search for the best method of rehabilitation triage at population level through a collaborative research initiative. From the state perspective, QEH is the main centre under JKNS that provides outreach rehabilitation medicine specialist care to all district hospitals throughout the state by conducting regular visiting clinics at set intervals. This service has expanded significantly since 2020 and by mid-2024 there were more than 15 visiting rehabilitation medicine specialist clinics in Sabah. This outreach service is confined to only rehabilitation physicians while depending on in-house therapists for frequent rehabilitation intervention sessions. At the society level, UMS has developed and initiated certification training for caregivers in community-based rehabilitation, targeting volunteers from the community as proxies to provide basic rehabilitation care to ensure uninterrupted treatment be programme is coupled with a comprehensive guide yond hospital. This training book written in collaboration between UMS and QEH.

### Collaborative research initiatives

Several studies investigating effective assessment methods and treatment modes have been undertaken and are ongoing between UMS and QEH in the field of rehabilitation medicine. A more significant research collaboration for capacity building in strengthening the health system for rehabilitation began in January 2024 with the aim to screen and evaluate clients in the primary care setting regarding their rehabilitation needs, ensuring early identification and accessibility to this service across all diseases and conditions. HUMS intends to evaluate the right method for triaging the population in the primary care setting for rehabilitation by proactively engaging renowned researchers involved in “ClinFIT” as a universal functioning information tool that can be utilized in the community setting (12). ClinFIT, short for Clinical Functioning Information Tool, is a standardized assessment system designed to measure an individual’s functional capacity across multiple domains. It is grounded in the International Classification of Functioning, Disability, and Health (ICF) framework, which provides a comprehensive view of health and disability. ClinFIT can be applied in both clinical and community settings, serving as a universal tool to assess functional outcomes and monitor changes over time. This tool is particularly useful in community-based settings as it enables healthcare providers to systematically evaluate physical, mental, and social functioning, which are critical for early intervention and holistic care. Clinically, ClinFIT allows for more individualized treatment planning by identifying specific functional limitations that might not otherwise be captured. For research, ClinFIT provides standardized, comparable data across diverse populations and contexts, enhancing the quality and generalizability of findings. The necessity for this specific tool is heightened by our experience of bringing the rehabilitation team to the community directly through the KRIS programme, in which we have identified a lack of awareness of rehabilitation among community members. These individuals lack understanding and access to rehabilitation services in the state. A screening method can ensure appropriate referrals to the rehabilitation team for effective assessment and interventions.

## DISCUSSION

The need to scale up rehabilitation is a critical call for action in Rehabilitation 2030. There is a need for global action by professional organizations, development agencies, and civil society to work towards developing and maintaining a sustainable workforce for rehabilitation. Viewed from the perspective of Sabah as the second largest state in the developing country of Malaysia, UMS, as the only public university in the state, has the social obligation to answer the health needs of its communities, particularly for those with functional limitations, by constructing capacity building in the field of physical and rehabilitation medicine in collaboration with JKNS. Effective collaboration between rehabilitation professionals and professional bodies in the state and Malaysia as a whole is essential to build a foundation framework for sustaining academic, research, and service capacity building. UMS, as a centre for education and academics, plays a pivotal role in helping establish the workforce required to widen and strengthen rehabilitation services qualities by building support with other relevant professional organizations and stakeholders, including JKNS.

**Fig. 2:**
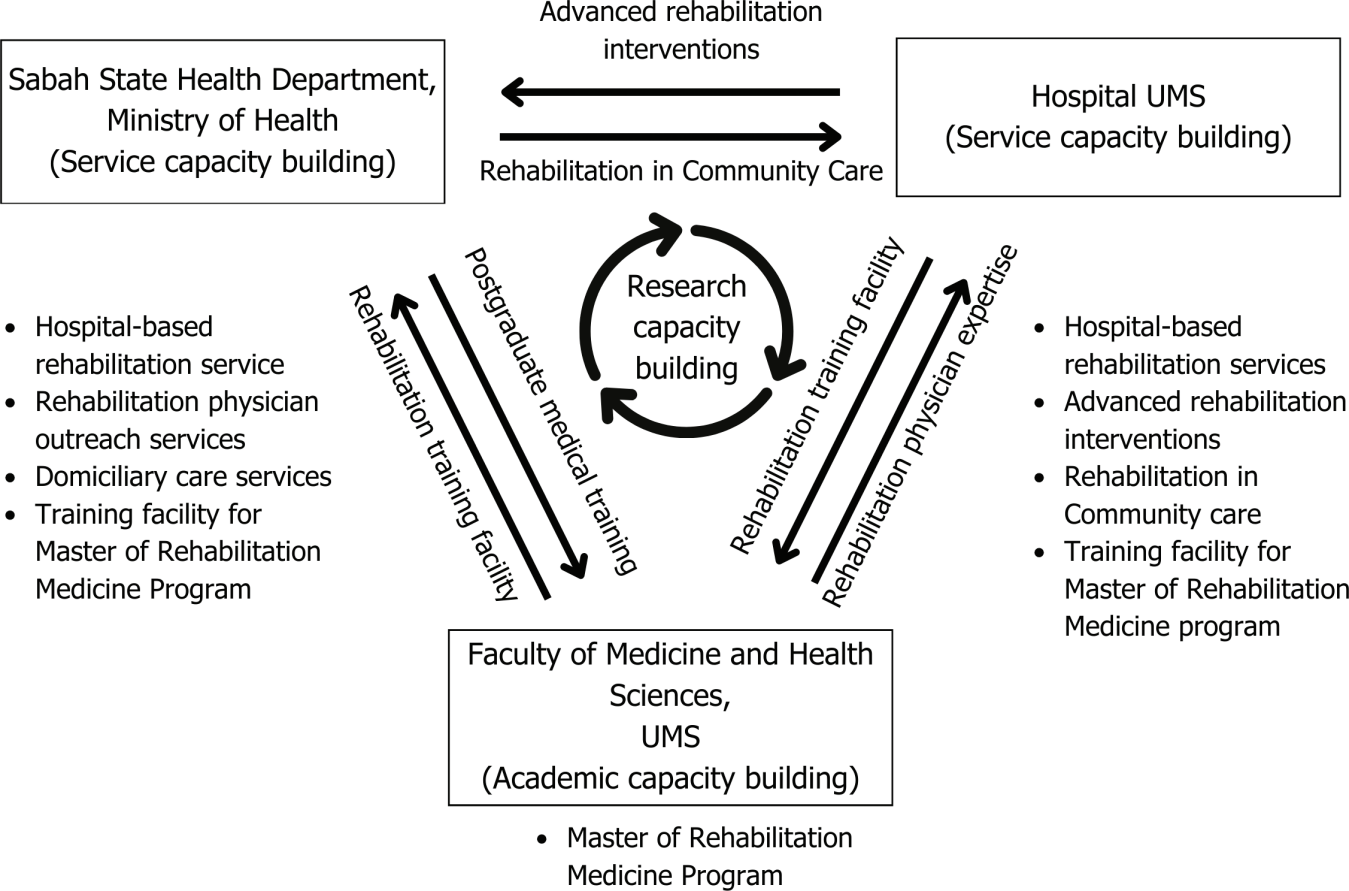
Foundation framework for strengthening rehabilitation in Sabah.

The strong collaboration between UMS and JKNS must be utilized strategically, to ensure strategic planning on the foundation framework to strengthen the health system for rehabilitation through capacity building materializes. Overall, the foundation framework is aimed at 3 perspectives (Fig. 2):

1. Academic capacity building: the European Academy of Rehabilitation Medicine (EARM) highlights several factors influencing academic capacity, including the values and goals of universities, educational needs and regulations, the scientific foundation of the field, health and social policies, governance structures, funding mechanisms, and the influence of organizations and individuals (2). In Malaysia, the Ministry of Higher Education (MOHE) is responsible for developing the required human resources through competency training and educational programmes. Universiti Malaysia Sabah (UMS), through its Faculty of Medicine and Health Sciences (FMHS), plays a pivotal role in this effort. The faculty is preparing to launch the country’s third postgraduate training programme for rehabilitation physicians, the Master of Rehabilitation Medicine, projected for intake in 2025/2026. This programme aims to increase the number of rehabilitation physicians in Sabah, with over 90% of candidates anticipated to come from the Sabah State Health Department (JKNS), which urgently needs to double its current capacity. Additionally, academic capacity must be expanded across all healthcare professionals’ curricula to enhance knowledge, awareness, early detection of rehabilitation needs, and timely referrals, ensuring that rehabilitation becomes an integral part of healthcare practice across all disciplines. The expansion aligns with Sabah’s plan to extend healthcare services to underserved areas, addressing the growing demand for specialized care.
2. Research capacity building: Establishing the first capacity building in rehabilitation medicine for Sabah presents a unique opportunity for UMS to collaborate with educational institutions globally. Beyond research, health information and data collection are also a management issue, forming one of the 6 essential building blocks of health systems. HUMS, through its Rehabilitation in Community Model, can serve as a model case for advancing functional assessment tools in the community using the ICF framework. This collaborative approach is exemplified by the Swiss Paraplegic Foundation, which aims to enhance the health and quality of life of individuals with spinal cord injury through clinical and community-based research via institutional networks (13). Similarly, Thailand’s research on ICF and community-based rehabilitation frameworks highlights the importance of identifying and addressing the needs of individuals with disabilities in rural communities (14). Such initiatives can only be realized through effective partnerships with JKNS’s hospitals and health clinics. Moreover, by developing a strong research foundation in rehabilitation at UMS, there is significant potential to build the necessary infrastructure, train researchers, and establish subspecialties, ultimately creating attractive career pathways for professionals in this field.
3. Service capacity building: it is essential that this working framework is not viewed as a physical and rehabilitation medicine (PRM) agenda exclusively, because without the cooperation of professional societies and allied healthcare professionals it will not be possible to create the advocacy energy required to build academic capacity to the point where it is sustainable (2). The Department of Rehabilitation Medicine of HUMS has a comprehensive team and advanced interventions to complement effective rehabilitation services along the continuum of care, from hospital care to the community. As the rehabilitation services of JKNS have catered excellently in offering care for bedbound patients through its Domiciliary Care Service, HUMS is able to complement the needs of the state through care via its Rehabilitation in Community Model.

Implementing capacity-building initiatives to strengthen rehabilitation services in Sabah would face several barriers and challenges. One of the primary challenges is resource constraints, particularly limited financial resources, which hinder the expansion and sustainability of rehabilitation programmes. Without adequate funding, procuring advanced rehabilitation equipment, expanding facilities, and providing training opportunities for healthcare professionals becomes difficult. Additionally, there is a significant workforce shortage, as the existing pool of rehabilitation specialists, including physiotherapists, occupational therapists, and speech therapists, is insufficient to meet the growing demands. This shortage not only affects service delivery but also limits opportunities for skill development and mentorship programmes essential for capacity building.

Geographical challenges further complicate the provision of rehabilitation services in rural and remote areas of Sabah, where access to care is often delayed due to transport difficulties and the lack of nearby facilities. These logistical issues exacerbate the workforce shortage, as healthcare professionals are less inclined to work in underserved regions. Cultural and language barriers, especially in indigenous communities, can also impede the effectiveness of rehabilitation interventions, making it essential to invest in culturally sensitive approaches and local community engagement. Addressing these challenges requires concerted efforts from both the state health department and university hospital, including securing long-term funding, improving workforce recruitment and retention strategies, and fostering partnerships to overcome geographical and cultural barriers.

The collaboration between HUMS and JKNS in developing rehabilitation services in Sabah has revealed key lessons that can guide future implementation. First, the need for a multidisciplinary approach is essential to meet the growing demands for rehabilitation, particularly in underserved and rural areas. The Rehabilitation in Community Model has shown the importance of bringing care closer to patients, but logistical barriers like transportation and workforce shortages must be addressed. Strengthening capacity building for training the rehabilitation workforce is critical, as the current pool of rehabilitation physicians, physiotherapists, occupational therapists, and other rehabilitation team members is insufficient to meet the needs of Sabah population. To overcome these challenges, strategic investments in workforce training, including postgraduate programmes and continuous professional development, are necessary. Empowering local communities through education and training initiatives will enhance the sustainability of rehabilitation services in remote areas.

Therefore, the next crucial step in these implementations is comprehensive monitoring and evaluation methods, which will be developed to track the effectiveness of these capacity-building initiatives. These methods will include key performance indicators (KPIs) to assess progress in areas such as training outcomes, service delivery, patient satisfaction, and overall improvements. Regular assessments and feedback mechanisms, including surveys and consultations with healthcare professionals, patients, and communities, will be employed to identify challenges and facilitate continuous improvement. However, the scope of this article does not yet cover findings from the monitoring and evaluation aspect, as these methods are still in the planning phase and will be implemented in the next stages of the initiative.

## CONCLUSION

The collaborative efforts between UMS and JKNS exemplify how academia and public health institutions can significantly influence policy development and resource allocation for rehabilitation services. Through academic programmes, UMS is addressing workforce shortages, directly impacting policy reforms to meet growing healthcare demands. Joint research initiatives contribute vital data to inform policy decisions, ensuring rehabilitation services are aligned with community needs. Furthermore, the integration of services fosters a stronger continuum of care, creating a foundation for sustained advocacy and strategic resource allocation to improve rehabilitation services in Sabah. This article intends that others may learn from our experiences in developing a working framework to strengthen rehabilitation capacity building in the Sabah context. By laying down this framework foundation, it must be viewed as part of a collaborative effort to implement strategic solutions for ensuring rehabilitation access throughout the state across the continuum of care. We welcome collaboration from other established models for academic capacity building and, more specifically, on research work for rehabilitation in the community. This is not a one-time call to action; however, we need ongoing dialogue, research, and action to drive lasting change.
